# Investigation of the Neutron Quantum States in the Earth’s Gravitational Field

**DOI:** 10.6028/jres.110.036

**Published:** 2005-06-01

**Authors:** V. V. Nesvizhevsky, A. K. Petukhov, H. G. Börner, T. A. Baranova, A. M. Gagarski, G. A. Petrov, K. V. Protasov, A. Yu. Voronin, S. Baeßler, H. Abele, A. Westphal, L. Lucovac

**Affiliations:** ILL, 6 rue Jules Horowitz, Grenoble, France; PNPI, Orlova Roscha, Gatchina, Leningrad reg., Russia; LPSC, 53 avenue des Martyrs, Grenoble, France; LPI, 53 Lenin prospect, Moscow, Russia; University of Mainz, 21 Saarstr., Germany; University of Heidelberg, 1 Grabengasse, Germany; DESY, Hamburg, Germany; UJF, BP 53, Grenoble, France

**Keywords:** gravitation, neutrons, quantum mechanics

## Abstract

We studied the neutron quantum states in the potential well formed by the Earth’s gravitational field and a horizontal mirror. The estimated characteristic sizes of the neutron wave functions in two lowest quantum states correspond to their expectations with an accuracy of ≈25 %. The spatial density distribution in a standing neutron wave above a mirror was measured for a set of a few lowest quantum states. A position-sensitive neutron detector with an extra high spatial resolution of 1 μm to 2 μm was developed and tested for this particular task. Although this experiment was not designed or optimized to search for an additional short-range force, nevertheless it allowed us to slightly improve the published boundary in the nanometer range of characteristic distances. We studied systematical uncertainties in the chosen “flow-through” method as well as the feasibility to improve further the accuracy in this experiment.

## 1. Introduction

Quantum states of a particle with mass *m* above an ideal horizontal mirror in the Earth’s gravitational field with the acceleration *g* are described by the Schrödinger equation, which is solved analytically in textbooks on quantum mechanics [1]. A possibility to measure such states for neutrons was discussed in Ref. [2]. The lowest quantum state of neutrons in such a system was observed in a recent experiment at the Institute Laue-Langevin [3]. The energy values and the neutron wave functions in such a system depend on *m*, *g*, on the Planck constant and on the quantum number *n* only. A mirror can be approximated as an infinitely high and sharp potential step compared to other characteristic parameters of the problem. Note that the neutron energy in the lowest quantum state ≈10^−12^ eV is much lower than the effective potential of a mirror ≈10^−7^ eV, and the range of increase of this effective potential ≈1 nm is much shorter than the neutron wavelength in the lowest quantum state ≈10 μm.

In the classical mechanics, a neutron with an energy *E_n_* in a gravitational field can rise to the maximum height of *z_n_* = *E_n_*/*mg*. In the quantum mechanics, a probability to observe a neutron in *n*th quantum state with an energy *E_n_* at a height *z* equals the square of the modulus of its wave function |*ψ_n_* (*z*)|^2^ in this quantum state. Formally, this value is not equal zero at any height *z* > 0. However, as soon as a height *z* is bigger than the characteristic size *z_n_* of the corresponding wave function, specific for every *n*th quantum state and precisely equal to the height of the neutron classical turning point, then a probability to observe a neutron approaches zero exponentially fast. Such a purely quantum effect of penetration of neutrons to a classically prohibited region is called the tunneling effect.

Such a wave-function shape allowed us to find a method to observe the neutron quantum states: to measure their transmission through a narrow slit Δ*z* between a horizontal mirror on bottom and a scatterer/absorber on top (referred to as a scatterer hereafter). If a scatterer is much higher than the turning point for the corresponding quantum state Δ*z* ≫ *z_n_*, then neutrons pass such a slit without significant losses. As a slit size decreases, the neutron wave function *ψ_n_*(*z*) starts touching the scatterer and the probability of neutron losses increases. If a slit size is smaller then the characteristic size of the neutron wave function in the lowest quantum state *z*_1_, then such a slit is not transparent for neutrons. Just this phenomenon was measured in our previous experiment [3]: the neutron flux through such a slit was equal zero if the slit size was smaller than 14 μm.

## 2. Experimental Installation

**Fig. 1:**
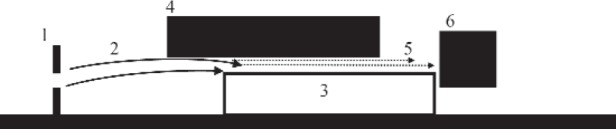
Principle of the present experiment. One can find from the left to the right the following: the vertical bold lines indicate the upper and lower plates of the input collimator (1); the solid arrows correspond to classical neutron trajectories (2) between the input collimator and the entrance slit between a mirror (3, the empty rectangle below) and a scatterer (4, the black rectangle above). The dotted horizontal arrows illustrate the quantum motion of neutrons above a mirror (5), and the black box presents a neutron detector (6). The size of the slit between a mirror and a scatterer could be varied and measured.

The experimental installation and the method of measurement are analogous to those used in our previous work [3] (see Fig. 1). We measure the neutron flux through a slit between a mirror and a scatterer versus the slit size. The slit size could be finely adjusted and precisely measured. The spectrum of the neutron horizontal velocity components is shaped by the input collimator with two plates, each of which could be adjusted independently to a required height. A low-background detector measures the neutron flux at the spectrometer exit. Ideally, the vertical and horizontal neutron motions are independent. This is valid if neutrons are reflected specularly from a horizontal mirror and if any influence of a scatterer, or that of any other force, is negligible to those neutrons which penetrate through the slit. If so, the horizontal motion of neutrons (with an average velocity of ≈5 m/s) is ruled by the classical laws, while in the vertical direction one observes the quantum motion with an effective velocity of a few centimeters per second and with a corresponding energy of a few peV (10^−12^ eV). The degree of validity of each condition is not evident a priori and should be verified in respective experiments. Thus, the efficiency of scatterers was shown to be close to unity and the probability of non-specular reflection of neutrons from bottom mirrors was measured to be at least as low as ~1 %. The absolute distance between a mirror and a scatterer was measured using a “capacitor” method.

## 3. Description of Experimental Data

Our installation is a precision one-component gravitational neutron spectrometer. The most important limit for its spatial resolution in the mode of scanning the neutron density with a scatterer follows from the finite sharpness of the gravitational barrier penetrability in function of a scatterer height. Our model is analogous to the well known model of nuclear *α*-decay: the neutron loss rate is proportional to the probability of neutron tunneling through the gravitational barrier between the classically allowed region and the scatterer height multiplied by the frequency of neutron collisions with the gravitational barrier. Detailed justification of this approach will be given in a forthcoming publication. At the moment let’s just present the parameterization, which is used to analyze the experimental data within framework of this model:

$$F(\Delta z,V_{\text{hor}})=\sum_{n}F_{n}(\Delta z,V_{\text{hor}})=\sum_{n}\left(\beta_{n}\cdot \exp\left\{-\alpha \cdot \frac{L}{V_{\text{hor}}}\cdot \exp\left[-\frac{4}{3}\cdot {\left(\frac{\Delta z-z_{n}}{z_{0}}\right)}^{\frac{3}{2}}\right]\right\}\right),$$ (1)

where *F*(Δ*z*,*V*_hor_) is the neutron flux through the slit, *α* is the coefficient responsible for a finite scatterer efficiency and for the approximations used in the model, *β_n_* is a population of *n*th quantum state, *V*_hor_ is a neutron horizontal velocity component, and *L* is the mirror length along the neutron beam axis. One can show that a scatterer does not disturb too much the neutron wave functions and that allows us to extract the values of non-disturbed critical heights *z_n_* with an uncertainty of about ±1.5 μm.

**Fig. 2:**
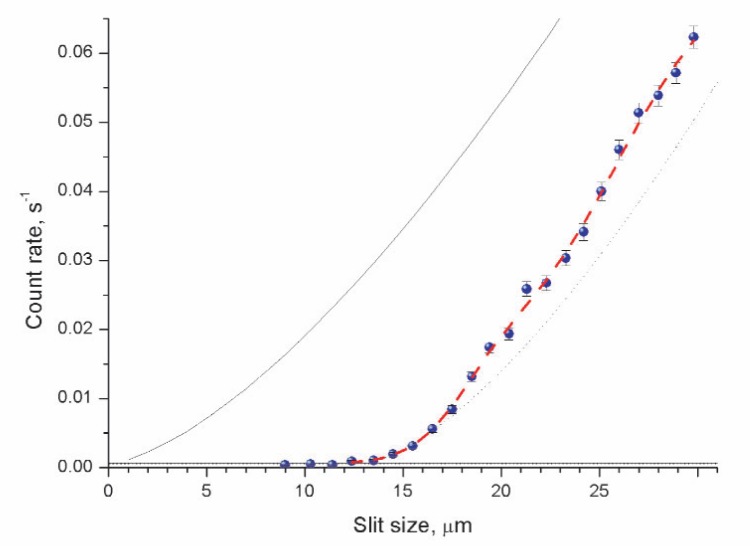
The dependence of the neutron flux through a slit between a mirror and a scatterer versus the slit size. The circles show the experimental results. The solid curve corresponds to the classical expectation normalized so that it approximates the experimental data at large slit sizes. The dotted line illustrates a simplified quantum-mechanical dependence, which assumes an existence of the lowest quantum state alone and the classical asymptotics at big slit sizes. The horizontal lines indicate the detector background and its uncertainty. The average horizontal neutron velocity component along the neutron beam axis is equal to 4.9±0.2 m/s. The dashed curve approximates the experimental data with a quantum-mechanical dependence [Eq. (1)].

The dependence of the neutron flux through a slit between a mirror and a scatterer versus the slit size was measured in an analogous way to that in Ref. [3], with better accuracy of the optical elements positioning and with higher statistical accuracy. Besides, we studied here in more details the dependence of the neutron flux *F*(Δ*z*,*V*_hor_) [Eq. (1)] on the value of a horizontal neutron velocity component as well as on a type of scatterer. We present here just one set of measurements on Fig. 2.

The curve (2) in Fig. 2 approximates the experimental data with *χ*^2^/*dof* = 0.9. In this calculation, the critical heights {*z_n_*, *n* > 2} corresponded to the above mentioned values for pure quantum states while the two lowest quantum heights *z*_1_ and z_2_ were fitted from the experimental data; the quantum state populations {*β_n_*, *n* > 1} were supposed to be equal except for the lowest quantum state population *β*_1_, which was calculated from the experimental data; the scatterer efficiency *α* was defined from the data as well.

Comparison of this model with the experimental data shows that: 1) the lowest quantum state population (*β*_1_ ≈ 0.7 ± 0.1) is lower than populations of higher quantum states as this was measured as well in Ref. [3]; 2) first and second critical heights *z*_1_ and *z*_2_ (taking into account an actual spatial resolution of the method) are equal to $\left(z_{1}^{\exp}=12.2\pm 1.8_{\text{syst}}\pm 0.7_{\text{stat}}\;\mu m\right)$ and $\left(z_{2}^{\exp}=21.3\pm 2.2_{\text{syst}}\pm 0.7_{\text{stat}}\;\mu m\right)$, which do not contradict to the expected quasi-classical values: $\left(z_{1}^{\text{theor}}=13.7\;\mu m\right)$ and $\left(z_{2}^{\text{theor}}=24.0\;\mu m\right)$ within 25 %.

## 4. Measurement of the Neutron Spatial Density Distribution Using a Position-Sensitive Detector

A direct measurement of the spatial density distribution in the neutron standing wave above a mirror is preferable compared to its study using a scatterer with a variable height. This so-called differential method is much more sensitive. Besides, a scatterer (used in the integral method) unavoidably disturbs the measured quantum states: it deforms the wave functions and shifts the energy levels. A finite accuracy of the corresponding corrections causes systematic uncertainties, which finally limit an achievable accuracy of measurement of the quantum state’s parameters. These reasons make attractive the application of a position-sensitive detector in order to directly measure the probability to observe neutrons above a mirror. We developed such a detector and the method of measurement: a plastic track nuclear detector (CR39) with a uranium coating (^235^UF_4_) described in Ref. [3]. The point of entrance of a neutron into the plastic detector was measured, after its chemical treatment, using an optical microscope that allows one to scan the detector surface along the distance of about 10 cm with an accuracy of ≈1 μm.

**Fig. 3:**
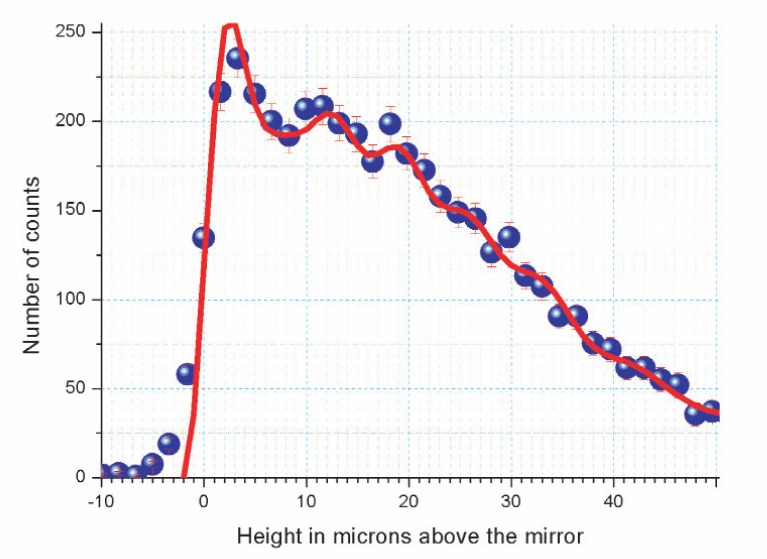
Preliminary results of a measurement of the neutron density above a mirror in the Earth’s gravitational field are obtained using a high-resolution plastic nuclear-track detector with a uranium coating. The horizontal axis corresponds to a height above a mirror in μm. The vertical axis gives the number of events in an interval of heights. The solid line shows the theoretical expectation under the assumption that the spatial resolution is infinitely high. Calculated populations of the quantum states are equal to those measured by means of two scatterers using the method.

The present test was aimed to estimate the spatial resolution of such a detector, so it was not optimized for an investigation of the phenomenon itself of the quantum states of neutrons above a mirror. In particular, we measured a few quantum states simultaneously in order to increase statistics and sharpness of increase of neutron count rate at zero height above mirror. Preliminary results obtained with this detector are shown in Fig. 3, where: 1) the neutron wave functions in the quantum states are known; 2) the calculated populations of the quantum states are equal to those measured with the method of two scatterers (this will be described in a forthcoming paper: the first scatterer shapes the neutron spectrum, the second scatterer measures the spectrum after some storage in the quantum states); 3) the zero height is estimated from the experimental data; 4) the difference between the idealized theoretical curve and the experimental results corresponds to a detector spatial resolution as high as ≈1.5 μm. Even a relatively small variation of the neutron density of about 10 % (expected for a few quantum states) could be measured in such a way. This means that selection of one-two quantum states would allow us to easily identify and measure them.

## 5. Outlook

In the present work, we investigated the quantization of neutron states in the potential well formed by the Earth’s gravitational field and a horizontal mirror. The conclusion of work [3] about presence of such a quantization is confirmed with higher statistical accuracy and methodical reliability. The measured characteristic sizes of the lowest two neutron wave functions *z*_1_ and *z*_2_ agree within an accuracy of ≈25 % with the expected values. The accuracy of this experiment is defined by its systematical uncertainty (the scatterer positioning and the model uncertainties). Systematic errors of the present method, as well as the ways to decrease them, were investigated. We showed that the spatial resolution of the method is close to its theoretical limit, defined by the sharpness of the neutron wave functions versus height, or, in other words, by the sharpness of the gravitational barrier permeability for neutrons versus height. The accuracy of this experiment could be improved by a few times if a more precise model of neutron interaction with a scatterer could be developed and the scatterer positioning accuracy could be improved. More significant increase in the accuracy of an experiment of such a kind could be achieved by means of long storage of neutrons in quantum states and by a measurement of the frequency of resonant transitions between them, which would allow one to calculate directly the energies of the corresponding quantum states.
